# Discovery of a Necroptosis Inhibitor Improving Dopaminergic Neuronal Loss after MPTP Exposure in Mice

**DOI:** 10.3390/ijms22105289

**Published:** 2021-05-18

**Authors:** Sara R. Oliveira, Pedro A. Dionísio, Maria M. Gaspar, Maria B. T. Ferreira, Catarina A. B. Rodrigues, Rita G. Pereira, Mónica S. Estevão, Maria J. Perry, Rui Moreira, Carlos A. M. Afonso, Joana D. Amaral, Cecília M. P. Rodrigues

**Affiliations:** Research Institute for Medicines (iMed.ULisboa), Faculty of Pharmacy, Universidade de Lisboa, 1649-003 Lisbon, Portugal; sararoliveira@ff.ulisboa.pt (S.R.O.); pedelandionisio@gmail.com (P.A.D.); mgaspar@ff.ulisboa.pt (M.M.G.); mariabtferreira@gmail.com (M.B.T.F.); cataxana@gmail.com (C.A.B.R.); ragpereira@gmail.com (R.G.P.); monica.estevao@gmail.com (M.S.E.); mjprocha@ff.ulisboa.pt (M.J.P.); rmoreira@ff.ulisboa.pt (R.M.); carlosafonso@ff.ulisboa.pt (C.A.M.A.); jamaral@ff.ulisboa.pt (J.D.A.)

**Keywords:** MPTP, necroptosis, neurodegeneration, Parkinson’s disease, small molecules

## Abstract

Parkinson’s disease (PD) is the second most common neurodegenerative disorder, mainly characterized by motor deficits correlated with progressive dopaminergic neuronal loss in the substantia nigra pars compacta (SN). Necroptosis is a caspase-independent form of regulated cell death mediated by the concerted action of receptor-interacting protein 3 (RIP3) and the pseudokinase mixed lineage domain-like protein (MLKL). It is also usually dependent on RIP1 kinase activity, influenced by further cellular clues. Importantly, necroptosis appears to be strongly linked to several neurodegenerative diseases, including PD. Here, we aimed at identifying novel chemical inhibitors of necroptosis in a PD-mimicking model, by conducting a two-step screening. Firstly, we phenotypically screened a library of 31 small molecules using a cellular model of necroptosis and, thereafter, the hit compound effect was validated in vivo in a sub-acute 1-methyl-1-4-phenyl-1,2,3,6-tetrahydropyridine hydrochloride (MPTP) PD-related mouse model. From the initial compounds, we identified one hit—Oxa12—that strongly inhibited necroptosis induced by the pan-caspase inhibitor zVAD-fmk in the BV2 murine microglia cell line. More importantly, mice exposed to MPTP and further treated with Oxa12 showed protection against MPTP-induced dopaminergic neuronal loss in the SN and striatum. In conclusion, we identified Oxa12 as a hit compound that represents a new chemotype to tackle necroptosis. Oxa12 displays in vivo effects, making this compound a drug candidate for further optimization to attenuate PD pathogenesis.

## 1. Introduction

Parkinson’s disease (PD) is the second most common neurodegenerative disorder worldwide. PD is pathologically defined by the progressive dysfunction of the nigrostriatal pathway, which culminates in the loss of dopaminergic neurons in the substantia nigra pars compacta (SN) and depletion of dopaminergic enervation in the striatum [1,2]. Of note, degeneration of dopaminergic neurons in the SN precedes the first motor deficits afflicting PD patients [2].

The pathological mechanisms causing PD are thought to stimulate a cascade of events that activate regulated cell death (RCD) pathways, which are responsible for neuronal death [3,4]. Recent evidence has shown that necroptosis, a type of regulated necrosis, plays crucial pathogenic roles in several human diseases, including neurodegenerative diseases, such as PD, while holding high potential for clinical targeting [5]. Necroptosis is a caspase-independent type of RCD commonly executed after RIP1 and RIP3 kinase activation. Typically, this type of cell death is initiated following cell death transmembrane receptor stimulation, with tumor necrosis factor (TNF) receptor 1 (TNFR1) being the most well-studied example [6,7]. In conditions where caspase-8 activation is genetically or pharmacologically prevented, RIP1 and RIP3 are not cleaved and accumulate in the so-called necrosome complex [8]. Then, activated RIP3 recruits and phosphorylates pseudokinase mixed lineage domain-like protein (MLKL), which further translocates to the plasma membrane, inducing membrane disruption and necroptosis execution [5]. Importantly, necroptosis has already been associated with neuronal death induced by the neurotoxin 1-methyl-4-phenyl-1,2,3,6-tetrahydropyridine (MPTP), a PD-mimicking neurotoxin, in both in vivo and in vitro rodent models, as well as in PD human samples [9,10].

Importantly, the first necroptosis inhibitors were identified back in 2005 through a phenotypic screening for chemical inhibitors of necroptosis induced by TNF and zVAD-fmk in human monocytic U937 cells. This led to the identification of necrostatin-1 (Nec-1) and its optimized derivative necrostatin-1 stable (Nec-1s), later confirmed to be RIP1 kinase inhibitors [11,12,13]. Additional studies regarding Nec-1 properties pointed to off-target activity and limited metabolic stability in mice, while Nec-1s presented higher activity as a necroptosis inhibitor, along with no nonspecific cytotoxicity and reasonable pharmacokinetic properties. However, Nec-1s still presents a short in vivo half-life [11,14,15,16,17]. Of note, Nec-1s identification proved that RIP1 inhibition is beneficial in several diseases [9,10,13,18]. Moreover, molecules targeting other components of necroptotic signaling pathway, such as RIP3 or MLKL, have also been proposed [19,20]. However, none of the compounds discovered so far reached the expectation of clinical application, which has led researchers to unceasingly screen new drugs with better selectivity and potency.

In this study, we phenotypically screened a library of 31 new molecules to discover novel inhibitors of necroptotic cell death with structural novelty. Although target-based drug discovery has been the dominant approach to drug discovery in the past decades, there has been a recent reawakening interest in phenotypic drug discovery approaches, based on their potential to address the complexity of only partially understood diseases and their promise of delivering first-in-class drugs, in parallel with major advances in cell-based phenotypic screening tools. In addition, prioritized hits from the primary screen were further evaluated for in vivo proof-of-concept using an MPTP PD-mimicking mouse model. We identified a potential hit, Oxa12, that significantly inhibited zVAD-fmk-induced necroptotic cell death in the BV2 microglial cell line, with a half-maximal effective concentration (EC_50_) of ~1 µM. Importantly, Oxa12 showed to protect dopaminergic neuronal cells from death induced by MPTP in vivo, in the SN and striatum, which highlights the potential benefits of discovering and optimizing new molecules with mechanisms of action that affect disease-relevant pathways, such as necroptosis.

## 2. Results

### 2.1. Phenotypic Screening for Hit Selection

**Figure 1:**
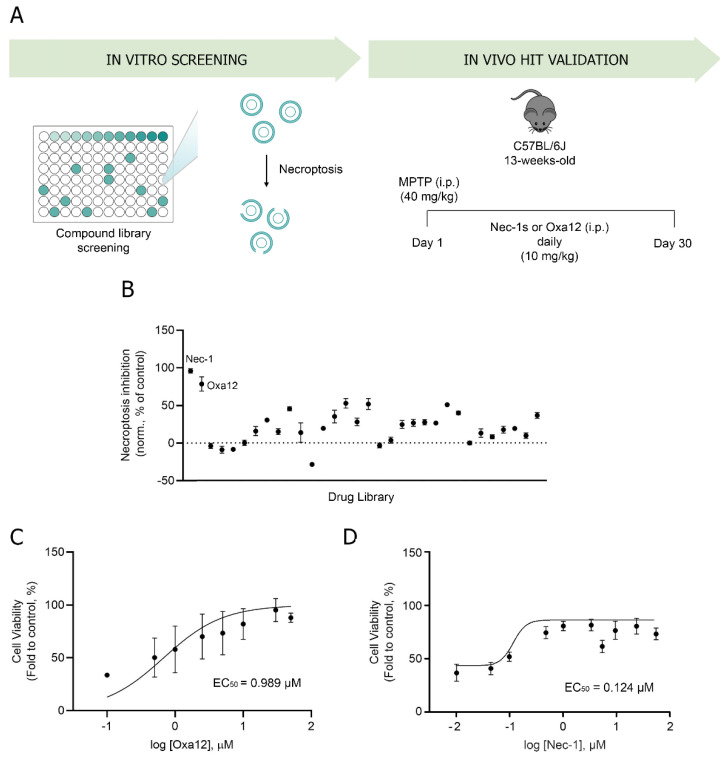
A cell-based phenotypic screening identifies Oxa12 as a necroptosis inhibitor. (**A**) Schematic overview of the two-step screening workflow. (**B**) Determination of the ability of the compound to inhibit necroptosis. Cell metabolic activity is depicted as a percentage of the control (DMSO = 0; Nec-1 at 30 µM) for compounds at 30 µM tested in BV2 murine microglia cells exposed to 25 µM zVAD-fmk for 24 h. (**C**) Half-maximal effective concentration (EC_50_) determination in BV2 murine microglia cells in a dose-response concentration (0.1 to 50 µM Oxa12) plus 25 µM zVAD-fmk for 24 h. (**D**) Half-maximal effective concentration (EC_50_) determination in BV2 murine microglia cells in a dose-response concentration (0.01 to 50 µM Nec-1) plus 25 µM zVAD-fmk for 24 h.

To identify novel necroptosis inhibitors, we performed a cell-based phenotypic screening assay to select hit compounds that strongly inhibit necroptotic cell death (Figure 1A).

**Figure 2:**
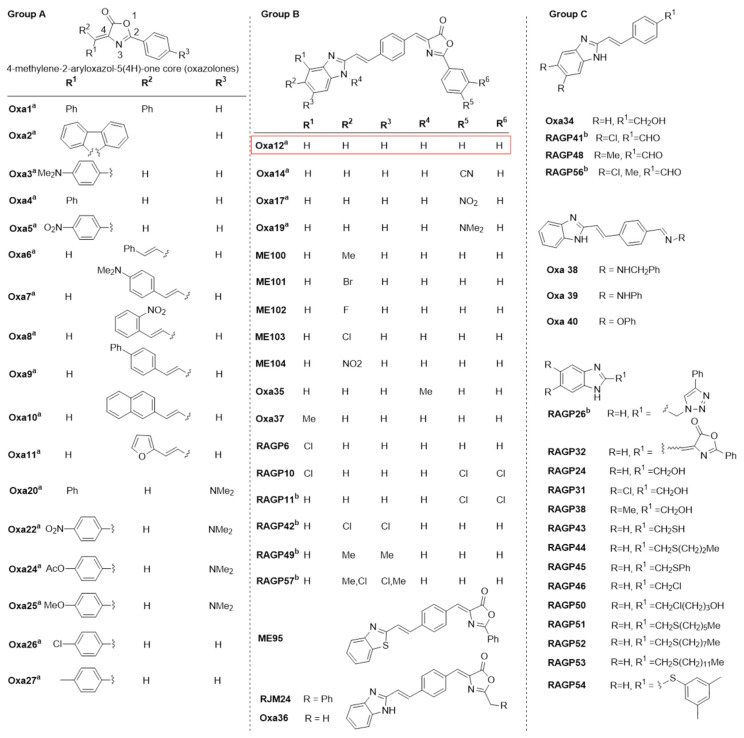
Structures of the synthesized small-molecule library compounds based on the 4-methylene-2-aryloxazol-5(4H)-one core (oxazolones). Group A includes oxazolones containing substituted aryl and aromatic heterocyclic attached to the 4-methylene position. Group B consists of extended oxazolones containing terminal fused heterocycles. Group C includes additional compounds without the oxazolone core or the 4-methylene side chain. ^a^ Synthesized compounds evaluated in a previous paper [22]. ^b^ Compounds not evaluated due to insoluble properties at the tested concentration.

Previous studies have demonstrated that the pan-caspase inhibitor zVAD-fmk induces necroptosis in different cellular models, including in the L929 fibrosarcoma and in the BV2 murine microglial cell lines, by a mechanism that most likely depends on TNF autocrine secretion [12,21,22]. Here, we used BV2 cells incubated with zVAD-fmk as an in vitro model of necroptosis and screened a total of 31 small compounds potentially inhibitors of this type of cell death. These compounds are part of a larger in-house library containing mainly the heterocyclic core of 4-methylene-2-aryloxazol-5(4H)-one and are represented in Figure 2. The remaining compounds have been tested in previous papers [12,21,22]. Cells were incubated with 25 µM zVAD-fmk alone or in combination with 30 µM test compounds for 24 h. BV2 cells treated with DMSO were used for data normalization. BV2 cells co-incubated with zVAD-fmk and Nec-1, a well-known RIP1 kinase inhibitor, were used as positive control of necroptosis inhibition (Figure 1B). As expected, treatment with Nec-1 almost completely rescued zVAD-fmk-induced decrease in cell viability (*p <* 0.001) (Figure 1B), as assessed by MTS metabolism. More importantly, we identified one hit compound—Oxa12—that significantly protected cells from zVAD-fmk-mediated necroptosis by ~70% (*p <* 0.01) (Figure 1B). Importantly, RIP1 kinase activity inhibitory potential of Oxa12 was determined using radiometric binding-based assays. The results show that Oxa12 at 5 µM inhibits RIP1 activity by ~15%, which confirms RIP1 as a possible target. The anti-necroptotic effects, however, may not be solely dependent on this target. These results are in line with our previous work, where we showed the sequestration of key necroptosis mediators, RIP1, RIP3, and p-MLKL, in the insoluble fraction in zVAD-fmk-treated BV2 cells, while Oxa12 abolished necrosome assembly and MLKL phosphorylation [22]. Moreover, Oxa12 strongly rescued zVAD-fmk-induced cell death, as observed by microscopy analysis of cell morphology [22]. Furthermore, our previous in silico molecular docking calculations for Oxa12 inside the RIP1 kinase domain demonstrated that Oxa12 is occupying a region similar to the co-crystallized inhibitor, suggesting a similar interaction pattern and indicating that Oxa12 most likely targets RIP1 to some extent [22].

To further characterize Oxa12 activity, we determined the EC_50_, a pharmacologic parameter commonly used as a measure of compound potency, which here indicates 50% of compound maximal potency to inhibit necroptosis, by performing a dose-response study. BV2 cells were incubated with zVAD-fmk along with Oxa12 at a range of concentrations (0.1–50 µM) for 24 h and the EC_50_ was calculated. Our results showed that Oxa12 presented an EC_50_ value of 0.989 µM, comparable with 0.124 µM of Nec-1, thus indicating its effectiveness as a necroptosis inhibitor (Figure 1C,D).

We next found that Oxa12 met stringent criteria on activity, chemical tractability and structural novelty, including a low chemical similarity with Nec-1 and its analogues, as measured by Tanimoto’s index. Moreover, we analyzed whether Oxa12 was included in the central nervous system (CNS) drug property space, using a CNS multiparameter optimization (MPO) approach, a prospective design tool and a widely utilized predictor for ADME and safety properties suitable for CNS targeting [23]. CNS MPO utilizes a set of six physicochemical descriptors to calculate a score, between 0 and 6. A higher score represents the optimal chemical space possessing alignment of key drug properties for CNS therapeutic agents. Generally, a CNS MPO score ≥ 4 is desired, although there are drugs active on the CNS with lower score, with a range of 3–4 being still acceptable [24]. Here, we observed that Oxa12 has a value of 3.6. While being lower than the CNS MPO score of 5 calculated for Nec-1, the value for Oxa12 is similar to that determined for several well-known CNS drugs, suggesting good brain exposure (Table 1).

Finally, we also determined the extent of Oxa12 metabolism in mouse liver microsomes. Using our medium-throughput drug metabolism platform, we observed that 20% of Oxa12 was detected by HPLC after incubation for 30 min in mouse liver microsomes, which corresponds to an estimated half-life of 13 min, a liability that negatively impacts the systemic and brain exposure to Oxa-12. These results allowed us to calculate a clearance Clint in vitro of 0.99 mL/min/mg protein and a predicted hepatic extraction ratio of 0.76. Of note, although the original Nec-1 has a half-life of < 5 min, chemical optimization of this compound has led to commonly used derivatives with a half-life of about 1 h in mouse microsomal assays [25]. Thus, a combined analysis of the CNS MPO score and metabolic data confirms that Oxa12 is a novel addition to the chemical toolbox of CNS-targeting anti-necroptotic compounds.

### 2.2. In Vivo Efficacy of Oxa12 in the Sub-Acute MPTP Mouse Model

To determine if our identified hit compound was effective in vivo, we used a sub-acute MPTP mouse model. MPTP is the most widely used neurotoxin to mimic PD-related nigrostriatal degeneration, since it can be easily administered systemically, and human exposure to MPTP has been associated with severe parkinsonism, which reinforces the relevance of MPTP animal models [26,27]. Mechanistically, MPTP can rapidly cross the blood–brain barrier and, once in the brain, it is mostly oxidized to an intermediate, which then diffuses to the extracellular space and converts to the active toxic metabolite, 1-methyl-4-phenylpyridinium (MPP^+^) [28,29]. Then, dopaminergic neurons selectively uptake MPP^+^. In mitochondria, MPP^+^ inhibits the complex I of the respiratory chain, leading to decreased ATP generation along with the production of reactive oxygen species (ROS), and ultimately cell death [29,30]. In this regard, several studies have already demonstrated that necroptosis is involved in neuronal death induced by MPTP and that Nec-1/Nec-1s administration is neuroprotective in multiple MPTP exposure regimens [9,10].

**Figure 3:**
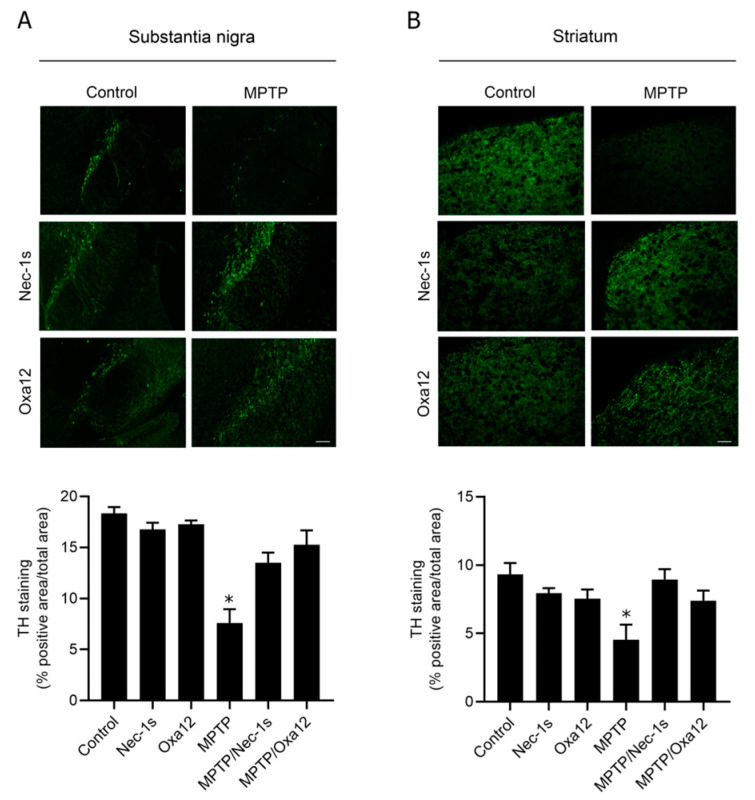
Oxa12 protects from MPTP-driven dopaminergic neuronal loss. Representative images of TH-positive immunostaining from control- and MPTP-injected mice treated with vehicle, Nec-1s or Oxa12 in the SN (**A**) and the striatum (**B**), and respective quantification. Scale bar, 100 µm. * *p* < 0.05 vs. control mice.

Here, we used a sub-acute MPTP regimen consisting of a single dose of MPTP (40 mg/kg), or vehicle, intraperitoneally injected in 13-week-old mice. Then, 10 mg/kg of Oxa12 or Nec-1s was administered 1 h after MPTP and then once every day for 30 days. As expected, we observed a significant reduction of approximately ~50% in TH-positive staining, a marker of dopaminergic neurons, in the SN and in the striatum of MPTP-exposed animals (Figure 3A,B), while no differences were observed in mice injected with Oxa12 or Nec-1s alone (Figure 3A,B).

Importantly, treatment with Oxa12 or Nec-1s partially protected cells from MPTP-induced dopaminergic cell death, in the SN (Oxa12, *p* = 0.05; Nec-1s, *p* = 0.12) and striatum (Oxa12, *p* = 0.06; Nec-1s, *p* = 0.08), as observed by the rescue of TH-positive staining in comparison with MPTP-treated animals (Figure 3A,B).

**Figure 4:**
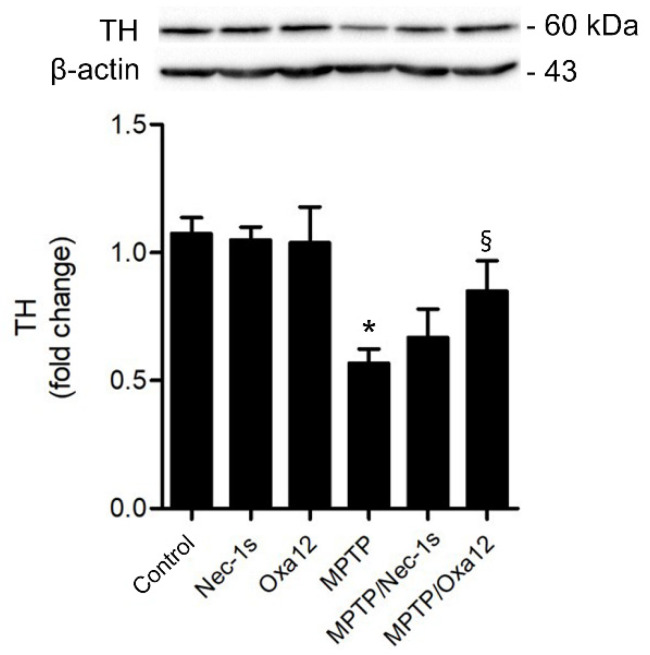
Oxa12 protects dopaminergic neurons from MPTP-induced cell death. Representative western blot of TH protein levels from control and MPTP-injected mice untreated or treated with Nec-1s or Oxa12 in the SN, and the respective densitometric analysis. β-actin was used as loading control. Values are expressed as mean ± SEM of three independent experiments. * *p <* 0.05 vs. control mice; § *p <* 0.05 vs. MPTP mice. TH, tyrosine hydroxylase.

In accordance, exposure to MPTP significantly reduced TH protein levels in the SN, while Oxa12 and Nec-1s treatment alone did not alter TH levels (Figure 4). Notably, Oxa12 significantly rescued TH protein levels by ~30% (*p* < 0.05), while Nec-1s showed a ~10% difference (Figure 4). The challenging dissection of SN-enriched midbrain sections, which include other dopaminergic structures, may account for the less significant rescue of TH in the SN by western blot as compared with immunofluorescence. Our results show that Oxa12 reveals a propensity to protect neuronal cells from MPTP-induced cell loss, with the potential to be explored as a new chemotype to tackle necroptosis. Nevertheless, careful examination of target engagement, including RIP1 and MLKL sequestration in insoluble fractions, a strong indicator of necrosome assembly and, therefore, of necroptosis commitment [28], deserves further investigation using appropriate antibodies.

Finally, MPTP mice models normally develop an inflammatory response that initiates in the striatum, with astrogliosis developing later than microgliosis and being sustained for a longer period of time [29,30]. Concordantly, we observed an increase in GFAP immunostaining and GFAP protein levels in MPTP-treated mice, thus suggesting a prolonged astrogliosis in these animals (data not shown). However, no significant differences were observed in mice treated with Oxa12 or Nec-1s.

## 3. Discussion

In the present study, a two-step screening workflow was performed to identify novel necroptosis inhibitors from a small-molecule library of 31 compounds. In a first step, we conducted an in vitro phenotypic screening to identify hit compounds that strongly inhibited zVAD-fmk-induced necroptotic cell death in the murine BV2 microglial cell line. In a second step, the ability of our hit molecule to protect against dopaminergic neuronal cell loss was determined in vivo using the sub-acute MPTP mouse model of PD.

As a strategy for the first step, we initially screened a range of diverse heterocyclic functionalized compounds collected from different developed synthetic methodologies. From this preliminary screening, several existing compounds containing the 4-methylene-2-aryloxazol-5(4H)-one (oxazolones) core were identified as bioactive [31]. Furthermore, we also synthesized a range of oxazolones containing diverse substituents at the 4-methylene and 2-aryl positions (Groups A and B) and by replacing the oxazolone core (Group C) as well as by inclusion of longer phenylmethylene spacer and additional structural tuning on the attached fused heterocycles (Group B). Importantly, we identified one molecule—Oxa12—that strongly inhibited necroptosis in the zVAD-fmk-treated BV2 microglia cells, and this effect was further demonstrated in vivo. Oxa12 tended to protect dopaminergic neuronal cells from MPTP-induced cell death, thus suggesting the potential of this compound in ameliorating PD pathogenesis.

Since the discovery of Nec-1 as the first necroptosis inhibitor, several other studies were performed and new inhibitors for RIP1, RIP3 and MLKL were identified. However, the therapeutic potential of all these inhibitors is restricted by low potency or reduced selectivity. In fact, so far, there is no compound that reached the prospects of clinical application, which has led to the continuous screening of new molecules with higher selectivity and potency. Despite its strongness at inhibiting necroptosis, Nec-1 is a far-from-ideal drug due to its short in vivo half-life of approximately 1 h, as well as its off-target effects, including inhibitory binding to indoleamine 2,3-dioxygenase (IDO), a protein involved in inflammation [32]. Nec-1s presents higher specificity and improved pharmacokinetics properties; however, it still has poor in vivo half-life [33]. Thus, Nec-1s represents a better choice for in vivo experiments. Other RIP1 inhibitors, including GSK’481, GSK’963 and GSK’772, demonstrate similar limitations [34,35,36]. RIP3 inhibitors, such as GSK’872, have also been studied; however, while they inhibit necroptosis, they may also promote apoptosis [20]. Thus, considering the limitations of all known RIP1 and RIP3 inhibitors, the development of novel and potent small compounds able to specifically attenuate necroptosis continues to be of strong need.

Here, we used the BV2 murine microglia cell line treated with the pan-caspase inhibitor, zVAD-fmk, as a model of necroptosis execution. In fact, we and others already demonstrated that zVAD-fmk strongly induces necroptotic cell death in both L929 fibrosarcoma and BV2 murine microglia cells [12,21,22]. Importantly, the pharmacological relevance of the in vitro phenotypic screening was guaranteed by using a well-established necroptosis inhibitor. Here, Nec-1, which inhibits RIP1 kinase activity, was used as positive control throughout the in vitro screening and demonstrated to be a strong necroptosis inhibitor. To identify novel necroptosis inhibitors, an in-house library of 31 small compounds were screened at a concentration of 30 µM, which allowed us to filter new necroptosis inhibitor scaffolds with potential to be further modified chemically. We identified one hit—Oxa12—that strongly inhibited necroptosis in the BV2 microglia cells by ~70%. To further characterize Oxa12 potency in vitro, dose-response curves were performed, where Oxa12 showed an EC_50_ value of 0.989 µM, thus reflecting its potential to be tested in vivo.

Throughout the years, MPTP mouse models have been widely used due to their specific and reproducible neurotoxic effect on the nigrostriatal system, being considered a convenient model of dopaminergic neurodegeneration to study therapeutic interventions. However, these models do not fully reproduce the human condition, and behavioral abnormalities are still a challenging question [36]. Moreover, previous studies have already demonstrated the involvement of necroptosis in the acute and sub-chronic MPTP mouse model of PD, where Nec-1/Nec-1s administration attenuated dopaminergic neurodegeneration [9,10]. Here, we used a sub-acute regimen of MPTP exposure, consisting of a single injection of MPTP (40 mg/kg) and still observed a significant increase in dopaminergic neurodegeneration in the SN and the striatum, while Nec-1s administration showed a tendency to protect cells from MPTP-induced dopaminergic cell death [27,37,38]. Importantly, Oxa12 alone did not induce neuronal toxicity, but it was able to protect dopaminergic neurons from MPTP-induced cell death in both SN and striatum. This experimental observation together with the MPO value suggest that Oxa-12 can cross the BBB, at least to some extent. Medicinal chemistry optimization of Oxa-12 should tackle issues such as solubility and be followed by extensive in vivo characterization of a more advanced lead compound, including by evaluating BBB penetration, half-life and target engagement. The role for inhibiting necroptosis deserves further exploitation [39].

Overall, these results indicate that Oxa12 has reasonable potency, associated with an apparent lack of toxicity, which makes this compound fit for additional chemistry optimization. Therapies using small molecules targeting key components of dopaminergic neuron cell death could evolve as a potential therapeutic approach for ameliorating PD progression.

## 4. Materials and Methods

### 4.1. Cell Culture and Reagents

The BV2 murine microglia cell line (gently provided by Dr. Elsa Rodrigues, University of Lisbon) was cultured in RPMI 1640 medium (GIBCO^®^ Life Technologies, Inc., Grand Island, NY, USA), supplemented with 10% heat inactivated fetal bovine serum (FBS), 1% antibiotic/antimycotic (A/A) solution and 1% GlutaMAX^TM^ (GIBCO). During the experiments, culture media was substituted by RPMI supplemented with 1% A/A, 1% insulin-transferrin-selenium (RPMI/ITS) and 1 mg/mL bovine serum albumin (BSA; GIBCO). Cells were maintained at 37 °C in a humidified atmosphere of 5% CO_2_. Chemicals used were Nec-1 (Sigma-Aldrich, St. Louis, MO, USA), dimethyl sulfoxide (DMSO; Sigma-Aldrich) and Z-Val-Ala-Asp-fluoromethylketone (zVAD-fmk) pan-caspase inhibitor (Enzo Life Sciences, Farmingdale, NY, USA).

### 4.2. Chemical Synthesis and Analysis

The oxazol-5-(4H)-ones (Oxas) and the precursors aldehydes were synthesized following a general synthetic reported route: a mixture of the corresponding aldehyde (30–600 µmol scale, 1 equiv.), hippuric acid or derivatives (1 equiv.), sodium acetate (1 equiv.) and acetic anhydride (3 equiv.) in a round-bottom flask was heated under magnetic stirring at 110 °C for 2 h [31]. Then, the reaction mixture was cooled to room temperature and the obtained solid was washed with distilled water (2×) and MeOH (4×) and dried under vacuum. If further purification was needed, some of the compounds were recrystallized in a suitable solvent. The purity and structural identification were performed by ^1^H, ^13^C NMR and mass spectrometry analyses.

### 4.3. Screening of Necroptosis Inhibitors

BV2 cells were plated in 96-well plates at 7 × 10^3^ cells/well and after 24 h, necroptosis was induced by adding 25 µM zVAD-fmk in RPMI/ITS. Test compounds or Nec-1 (positive control of necroptosis inhibition) were incubated along with zVAD-fmk at a final concentration of 30 µM for 24 h. DMSO was used as vehicle control. Cell viability was determined based on measurement of MTS metabolism using the CellTiter 96^®^ Aqueous Non-Radioactive Cell Proliferation (MTS) Assay (Promega, Madison, WI, USA). Differences in absorbance were measured at 490 nm using GloMax^®^ Multi Detection System (Sunnyvale, CA, USA).

### 4.4. EC_50_ Determination

To quantitatively determine the effective potency in BV2 cells, the compound half maximal effective concentration or EC_50_ was determined by a dose-response curve. In brief, BV2 cells were plated in 96-well plates as described above and treated with increasing concentrations of compound Oxa12 (from 0.1 to 50 µM) and Nec-1 (from 0.01 to 50 µM) using DMSO as vehicle control. Following 24 h of incubation, cell viability was determined based on the measurement of MTS, as previously described.

### 4.5. RIP1 and RIP3 Kinase Activity Assays

Oxa12 were tested at 5 μM for RIP1 kinase activity by a radiometric-binding assay using myelin basic protein as substrate (Eurofins, France) as before [39].

### 4.6. Microsomal Stability Assay

In the microsomal stability assay, an analytical high-performance liquid chromatography (HPLC) system was used with the following condition: column, Merck Lichrospher 100 RP18 125 mm 4.6 mm (5 µm); mobile phase A = 0.1% trifluoroacetic acid in water, B = 0.1% trifluoroacetic acid in acetonitrile, isocratic; flow rate, 1 mL/min; detection, UV at 400 nm injection, 20 µL; column temperature, ambient. The metabolic stability assays were carried out using the cosolvent method, appropriate for assessing the metabolic stability of compounds poorly soluble in aqueous medium [40]. For the cosolvent method, a 0.5 mM DMSO stock solution of Oxa12 was prepared. Then, a diluted solution of the compound was prepared by adding 50 µL of the previous 0.05 mM solution with 200 µL of acetonitrile, to make a 0.1 mM solution of Oxa12 in 20% DMSO/80% acetonitrile. Cosolvent assay conditions were: substrate concentration, 1 µM; microsomal protein, 0.5 mg/mL; organic solvents, 0.2% DMSO, 0.8% acetonitrile; incubation time, 30 min; number of assays, duplicates for T0 and T30 min. Time 0 and time 30 batches, after quenching with acetonitrile, were centrifuged at 11,000× *g* for 5 min and the supernatants were analyzed by HPLC, in order to quantify compound Oxa12.

### 4.7. MPTP Mouse Model

Animal studies were performed according to the animal welfare of the Faculty of Pharmacy, University of Lisbon, and approved by the competent national authority Direção-Geral de Alimentação e Veterinária (DAGV) and in accordance with the EU Directive (2010/63/UE), Portuguese laws (DR 113/2013, 2880/2015 and 260/2016) and all relevant legislation. To evaluate the neuroprotective effect of our hit compound—Oxa12—in the sub-acute MPTP mouse model, male 13-week-old C57BL/6N wild-type (wt) mice (Charles River Laboratories, Wilmington, MA, USA) were injected intraperitoneally (i.p.) with a unique dose of MPTP-HCl (40 mg/kg; Sigma Aldrich, St Louis, MO, USA), dissolved in sterile 0.9% saline, or vehicle only (control group). One hour after MPTP administration, mice were intraperitoneally injected with 10 mg/kg Oxa12 solubilized in 1% DMSO (Sigma-Aldrich) and 30% 2-hydroxypropyl-beta-cyclodextrin (Sigma-Aldrich) or 10 mg/kg Nec-1s (Focus Biomolecules, Plymouth Meeting, PA, USA) solubilized in 1% DMSO, 4% 2-hydroxypropyl-beta-cyclodextrin (Sigma-Aldrich), in PBS [38,41,42]. Oxa12 and Nec-1s injections were administered once every day for 30 days. Oxa12 dosage and regimen of administration were selected based on published protocols for Nec-1s [9,33]. Seven animal per group were used. After 30 days, mice were sacrificed in a CO_2_ chamber followed by transcardiac perfusion with ice-cold PBS. Brains were then excised, and one hemisphere was used to isolate the midbrain region, containing the SN, and the striatum, as previously described [38,41], which was rapidly frozen in liquid nitrogen and stored at −80 °C until processing for protein extraction. The other hemisphere was fixed in 4% paraformaldehyde for 48 h and then stored in 20% sucrose/PBS and 0.025% sodium azide, at 4 °C, for further immunohistochemistry analyses.

### 4.8. Immunohistochemistry

Hemispheres previously fixed in paraformaldehyde were cryoprotected in 20% sucrose/PBS and embedded in gelatin. Then, sequential coronal brain sections (8 µm thick) near the midstriatum (Bregma 1.00) and SN (Bregma −3.20) were obtained by cryostat sectioning and mounted on SuperFrost-Plus glass slides (Thermo Fisher Scientific). Afterward, sections were incubated in warm PBS at 37 °C for 15 min, followed by two washes in PBS, to remove gelatin. Then, sections were blocked in Tris-buffered saline (TBS) containing 10% (*v*/*v*) normal donkey serum (Jackson ImmunoResearch Laboratories Inc., West Grove, PA, USA) and 0.1% (*v*/*v*) Triton X-100 (Sigma-Aldrich) for 1 h. Subsequentially, to stain dopaminergic neurons, sections were incubated with primary rabbit polyclonal anti-tyrosine hydroxylase (TH) antibody (#ab112; Abcam, Cambridge, UK, 1:700), overnight at 4 °C. After several washes with PBS, anti-TH primary antibody was detected with diluted (1:200) Alexa Fluor 488 (anti-rabbit) conjugated secondary antibody (Invitrogen—Thermo Fisher Scientific) for 2 h at room temperature. After extensive rinsing, sections were counterstained with Hoechst 33258 (Sigma-Aldrich) and mounted on Mowiol 4-88 (Sigma-Aldrich).

### 4.9. Image Analysis

Images were obtained by an Axioskop fluorescence microscope (Carl Zeiss GmbH, Hamburg, Germany). Images were captured from six region-matched sections for nigral and striatal regions for each animal and converted into a gray scale with an 8-bit format using the ImageJ software version 1.48 (National Institute of Health, Bethesda, MD, USA). A threshold optical density was determined for each staining. Areas occupied by positive staining were quantified in thresholded images, normalized to the total area of interest region and calculated as percentage of total area.

### 4.10. Protein Isolation

For total protein isolation, tissues obtained from dissected midbrains and striata were homogenized in radio-immunoprecipitation assay (RIPA) buffer (50 nM Tris/HCl, pH 8; 150 nM NaCl; 1% NP-40; 0.5% sodium deoxycholate; 0.1% SDS) and 1x Halt Protease and Phosphatase Inhibitor Cocktail (Pierce, Thermo Fisher Scientific) with a moto-driven Bio-vortexer (No 1083; Biospec Products, Bartlesfield, UK). Lysates were maintained on ice for 30 min and were then sonicated and centrifuged at 10,000× *g* for 10 min. Supernatants were collected and used as total protein extracts. Protein concentrations were determined by using the Bio-Rad protein assay kit, according to the manufacturer’s instructions.

### 4.11. Western Blot

Equal amounts of total protein extracts were electrophoretically resolved on 8% SDS-PAGE. Resolved proteins were then transferred onto nitrocellulose membranes and blocked with a 5% milk solution in Tris-buffered saline (TBS). Then, membranes were incubated with primary antibody rabbit polyclonal TH (#ab112, Abcam), overnight at 4 °C. After washing with TBS/0.2% Tween 20 (TBS-T), membranes were incubated with secondary goat anti-rabbit IgG antibody conjugated with horseradish peroxidase (Bio-Rad Laboratories) for 2 h at room temperature. Membranes were processed for protein detection using Immobilon^TM^ Western (Millipore). β-actin (AC-15) (#A5441, Sigma-Aldrich) was used as loading control. Densitometric analysis was performed using the Image Lab Software version 5.1 Beta (Bio-Rad).

### 4.12. Statistical Analysis

All data are presented as mean ± standard error of the mean (SEM). Data analysis were performed with a one-way analysis of variance (ANOVA) followed by a post hoc Bonferroni test. Analysis and graphical presentation were conducted with the GraphPad Prim Software version 8 (GraphPad Software Inc., San Diego, CA, USA). Statistical significance was achieved when *p* < 0.05.

## Figures and Tables

**Table 1:** Central nervous system MPO calculations for Oxa12 and Nec-1.

Oxa12				Nec-1			
Physiochemical Descriptor		Individual Score	Score CNS MPO	Physiochemical Descriptor		Individual Score	Score CNS MPO
MW	391.43	0.8	3.6	MW	259.33	1.0	5.5
clogP	5.29	0		clogP	1.66	1.0	
clogD_7.4_	5.69	0		clogD_7.4_	1.66	1.0	
TPSA	67.34	1.0		TPSA	48.13	1.0	
HBD	1	0.8		HBD	2	0.5	
pK_a_	5.34	1.0		pK_a_	0	1.0	

MW, molecular weight; clogP, calculated logP (*n*-octanol/buffer distribution coefficient) using Molinspiration desktop property calculator (https://www.molinspiration.com/cgi-bin/properties, accessed on 15 April 2021); clogD_7.4_, distribution coefficient at pH 7.4; TPSA, topological polar surface area; HDB, number of hydrogen bond donor atoms; pK_a_, acidity constant of ionizable function.
