# Correction: Oliveira et al. Discovery of a Necroptosis Inhibitor Improving Dopaminergic Neuronal Loss after MPTP Exposure in Mice. *Int. J. Mol. Sci.* 2021, *22*, 5289

**DOI:** 10.3390/ijms26041612

**Published:** 2025-02-14

**Authors:** Sara R. Oliveira, Pedro A. Dionísio, Maria M. Gaspar, Maria B. T. Ferreira, Catarina A. B. Rodrigues, Rita G. Pereira, Mónica S. Estevão, Maria J. Perry, Rui Moreira, Carlos A. M. Afonso, Joana D. Amaral, Cecília M. P. Rodrigues

**Affiliations:** Research Institute for Medicines (iMed.ULisboa), Faculty of Pharmacy, Universidade de Lisboa, 1649-003 Lisbon, Portugal; sararoliveira@ff.ulisboa.pt (S.R.O.); pedelandionisio@gmail.com (P.A.D.); mgaspar@ff.ulisboa.pt (M.M.G.); mariabtferreira@gmail.com (M.B.T.F.); cataxana@gmail.com (C.A.B.R.); ragpereira@gmail.com (R.G.P.); monica.estevao@gmail.com (M.S.E.); mjprocha@ff.ulisboa.pt (M.J.P.); rmoreira@ff.ulisboa.pt (R.M.); carlosafonso@ff.ulisboa.pt (C.A.M.A.); jamaral@ff.ulisboa.pt (J.D.A.)

In the original publication [1], there was a mistake in Figure 3 as published. In Figure 3, panel A, representative images of tyrosine hydroxylase-positive immunostaining in substantia nigra from MPTP-injected mice treated with Nec-1s and Oxa-12 were wrongly selected in the original manuscript. The corrected Figure 3 appears below. The authors state that the scientific conclusions are unaffected. This correction was approved by the Academic Editor. The original publication has also been updated.

## Figures and Tables

**Figure 3:**
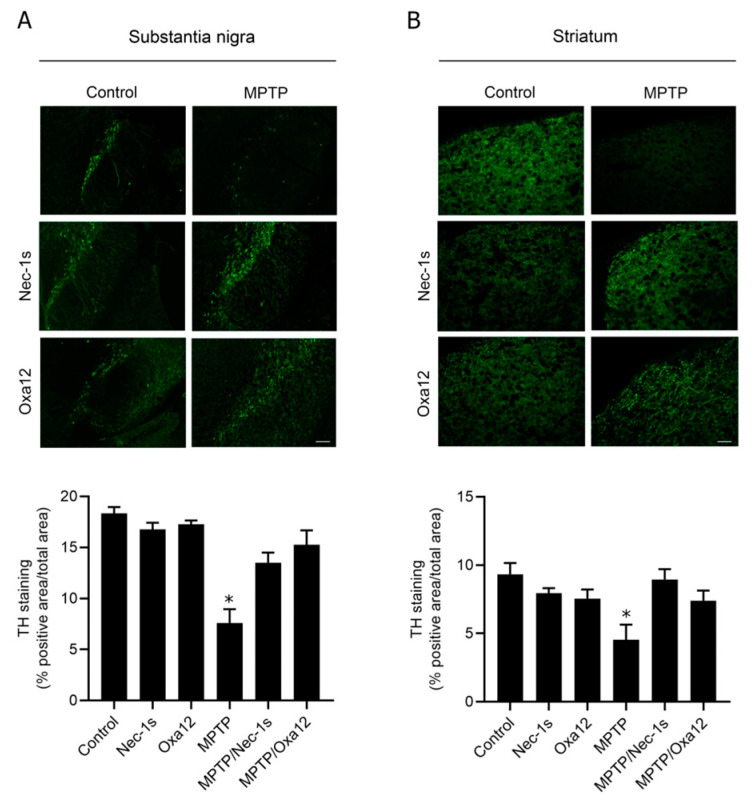
Oxa12 protects from MPTP-driven dopaminergic neuronal loss. Representative images of TH-positive immunostaining from control- and MPTP-injected mice treated with vehicle, Nec-1s or Oxa12 in the SN (**A**) and the striatum (**B**), and respective quantification. Scale bar, 100 µm. * *p* < 0.05 vs. control mice.
